# Behavior analysis and behavioral neuroscience

**DOI:** 10.3389/fnhum.2015.00210

**Published:** 2015-04-17

**Authors:** Henry D. Schlinger

**Affiliations:** Department of Psychology, California State UniversityLos Angeles, CA, USA

**Keywords:** behavior analysis, behavioral neuroscience, behavioral causation, adaptive behavior, operant conditioning

Behavior analysis—the science of adaptive behavior—focuses on behavior as a subject matter in its own right, not as an index of cognitive events, and is, thus, not dualistic. Behavior analysis incorporates several laws of learning discovered by researchers using single-subject experimental designs. I argue that behavior analysis can provide neuroscientists with an experimental and a theoretical framework within which to investigate the neural bases of behaviors, including those that are usually described in cognitive terms.

## The importance of behavior for neuroscience

Behavior includes anything an organism does whether it is observed or not. The emphasis on behavior should be appreciated within biopsychology given that behavior is a crucial evolutionary determinant of survival. It is what organisms do—for example, finding shelter, escaping predation, mating, or caring for offspring—that is important. As a result, the nervous system has evolved to meet the demands of interacting with and adapting to the environment. As Engel and Schneiderman (1984) noted, “the raison d'etre of the CNS is to optimize the organism's ability to interact with its environment” (p. 199).

Roughly speaking, the nervous system has evolved to carry out two functions related to an “organism's ability to interact with its environment”: detecting energy changes and controlling movement, with specific sensory and motor areas of the cortex devoted to each of these functions. Other cortical areas, however, are programmed largely by learning experiences (i.e., Pavlovian and operant conditioning). Research using Positron Emission Tomography (PET) scans that compares brain activity in newborns to that in older children and adults (e.g., Chugani et al., 1987; Chugani, 1999) has shown the most activity in the neonate's brain occurs in the primary sensory and motor cortexes, thalamus, and brainstem, areas associated with the primitive reflexes seen in infants. Activity in the frontal association cortex and other areas associated with “higher cortical and cognitive function” is relatively nonexistent. As infants interact with their environments, more activity is seen in areas of the cortex that mediate these behaviors. Such research supports the suggestion that learning is responsible for the significant changes in the brain related to complex behavior (Schlinger, 2004) and underscores the importance of behavioral plasticity.

The physical basis of behavioral plasticity is neuroplasticity; that is, interactions between an organism's behavior and its environment cause changes in the structure of the brain. There is a wealth of evidence of such changes in nonhumans (e.g., Turner and Greenough, 1985; Kolb and Whishaw, 1998; Rioult-Pedotti et al., 2000). Moreover, research shows that treatments based on operant conditioning can produce distinct changes in the human brain (e.g., Schwartz et al., 1996; Temple et al., 2003). To better investigate how the nervous system mediates adaptive behaviors, neuroscientists need to understand the functions of the behaviors themselves. Because organisms interact with their environment by behaving, then, “only when these organismic-environmental interactions are studied both behaviorally and physiologically, in a broad biological context, will it be possible to develop rational models of” behavioral causation (Engel and Schneiderman, 1984, p. 199).

## Levels of behavioral causation

Both evolutionary biologists (e.g., Mayr, 1988, 1997) and behavior analysts (e.g., Alessi, 1992) have classified behavioral causation in terms of ultimate and proximate causation. Ultimate causation answers *why* questions by describing the processes by which traits (analyzed by the sciences of proximate causation) evolved (Alessi, 1992, p. 1360). Ultimate causes are further classified as either phylogenetic or ontogenetic. The process of phylogenetic ultimate causation is natural selection, and is the domain of evolutionary biology. The processes of ontogenetic ultimate causation—Pavlovian and operant conditioning—are characterized by the selection of behavioral features of organisms during their lifetimes, and are the domain of behavior analysis (Skinner, 1981; Glenn et al., 1992). Proximate causation answers *how* questions and “is the domain of functional biology” (Alessi, 1992). At one level, functional biology is concerned with discovering *how* patterns of neuronal activity are translated into behavior. This enterprise is the domain of behavioral neuroscience.

Behavior is also caused proximately by changes in an organism's immediate environment. Stimuli that occur immediately prior to or contemporaneously with behavior are said to evoke the behavior (Schlinger and Blakely, 1994), but a more complete picture is that such stimuli evoke neural changes that, in turn, evoke behavior. For example, the patellar reflex is initiated by a tap on the patellar tendon, which causes a stretch receptor in the quadriceps muscle to fire. The sensory neuron synapses with a motor neuron in the lumbar region of the spinal cord, which sends a nerve impulse back to the quadriceps muscle causing it to contract, which is evident in leg extension. The proximate causes of the contraction of the quadriceps muscle are the stimulus (tap) and the sensory-motor nerve firing. The ultimate cause of the reflex lies in the evolutionary history of organisms in which it is found. Similarly, the question “What is two plus two?” initiates a chain of physiological events, which as proximate causes produce the behavior of saying “four.” The ultimate cause of this behavior lies in the operant learning history of the individual.

Said another way, ultimate causes establish proximate causes. For example, natural selection, as an ultimate cause, is responsible for genes, which as proximate causes produce proteins, the physical basis of the body including the brain and behavior. Likewise, (Pavlovian and operant) conditioning, as an ultimate cause, establishes and modifies both environmental stimuli *and* neural connections, which as proximate causes produce learned behavior. The structure of the brain, then, as a set of proximate causes for behavior, is co-determined by the ultimate causes of evolution by natural selection and conditioning. As Skinner (1990) explained, “Physiology studies the product of which the sciences of variation and selection study the production… [P]hysiology tells us *how* the body works; the sciences of variation and selection tell us *why* it is a body that works that way” (p. 1208). In an effort to understand behavior, then, behavior analysts explain *why* it occurs in terms of general laws, and neuroscientists explain *how* it occurs in terms of more elementary physiological processes. In order for neuroscientists to fully understand *how* learned behavior occurs (i.e., its proximate physiological causes), they must first understand *why* it occurs. In other words, neuroscientists need a theory of ultimate ontogenetic causation. Otherwise, they risk simply producing a vast taxonomy of unrelated neurophysiological functions. As Skinner (1938) wrote,

The discovery of neurological facts may proceed independently of a science of behavior if the facts are directly observed as structural and functional changes in tissue, but before such a fact may be shown to account for a fact of behavior, both must be quantitatively described and shown to correspond in all their properties (p. 422).

From a behavior-analytic perspective, then, behavioral neuroscientists examine the neurophysiological processes that underlie established functional relationships between behavior and environment. As such, neuroscientists will eventually be able to explain *how* behavioral processes (e.g., reinforcement, discrimination) work, and *how* environment-behavior relationships are established by conditioning and represented in the nervous system. That is, the behavioral laws can provide a road map for neuroscientists. As Donahoe and Palmer (1994) wrote, “analyses at the behavioral level define the boundaries within which the underlying physiological mechanisms must operate” (p. 54).

## The neural bases of operant conditioning

One advantage of a behavior-analytic approach, which stresses single-subject experimental methodology, is the elegant control (i.e., influencing directly the behavior of the single organism without relying on aggregate measures) that it affords. Substantial research already demonstrates how such experimental control can elucidate the neurophysical foundations of behavior. Beginning with the groundbreaking work by Olds and Milner (1954) showing that electrical brain stimulation in certain neural pathways could function as a powerful reinforcer, neuroscientists have identified many of the quantitative, anatomical, and physiological properties of those neural pathways (e.g., Gallistel, 1988; Hoebel, 1988). For example, research has shown that the most reliable location of reinforcing electrical brain stimulation is a bundle of axons, called the medial forebrain bundle, that travel from the ventral tegmental area (VTA) of the midbrain to the forebrain (Olds and Forbes, 1981). Moreover, dopaminergic neurons in the VTA and forebrain are primarily involved in the reinforcement of operant behavior (Hoebel, 1988).

The role of dopaminergic neurons in reinforcement is supported, in part, by findings showing that dopamine antagonists block the effects of natural reinforcers such as food, the effects of reinforcing electrical brain stimulation, and the effects of conditioned reinforcers (e.g., Franklin and McCoy, 1979; Gallistel and Karras, 1994). Conversely, dopamine agonists, such as amphetamine, function as powerful reinforcers whether they are injected into the blood stream, as in self-administration preparations, or directly into the brain, as in intracranial administration preparations (e.g., Hoebel et al., 1983; Guerin et al., 1984). Furthermore, electrical brain stimulation, dopamine agonists, and natural reinforcers all stimulate the release of dopamine in the mesolimbic and mesocortical systems (e.g., Moghaddam and Bunney, 1989; Nakahara et al., 1989), suggesting that the physiological mechanism of these different reinforcing events is the same.

Research has also revealed the possible cellular bases of operant conditioning by showing that individual neurons can be operantly conditioned (e.g., Belluzzi and Stein, 1983; Stein and Belluzzi, 1985). Other research has shown that operant conditioning can alter the structure of the brain, to include (1) regulating the dynamics of neuronal activity (Nargeot et al., 1999a), (2) changing synaptic terminals on primate motor neurons (Feng-Chen and Wolpaw, 1996), (3) altering dendritic branching and spine densities of CA3 pyramidal neurons of the hippocampus (Mahajan and Desiraju, 1988), and reorganizing the cerebral cortex (Bao et al., 2001).

Research on synaptic changes due to operant conditioning, as well as the susceptibility of individual neurons to operant conditioning, suggests that, “the individual neuron could be an important functional unit for positive reinforcement in the brain” (Stein and Belluzzi, 1988, p. 261). If so, such findings may illuminate “the neuronal substrate that underlies the selective modification in operant conditioning” (Nargeot et al., 1999b), and may help to persuade other neuroscientists that behavior analysis offers both a fruitful theory of behavior and a scientific methodology within which to better understand their findings, a guide for future research, and, hence, a more unified scientific understanding of behavior.

## Implications

Ironically, a science that deals with an objective and measurable subject matter—behavior—may offer neuroscientists a more productive theoretical model with regard to so-called cognitive events than an approach (i.e., cognitive science) that deals only indirectly with its subject matter. For example, neuroscientific evidence supports an interpretation of listening (Schlinger, 2008) and auditory imagining (Schlinger, 2009) as operant behavior. Studies using transcranial magnetic stimulation have shown that there is in increase in motor-evoked potentials recorded from the tongue muscles (Fadiga et al., 2002) and from the lip muscles (Watkins et al., 2003) during speech perception, that is, when someone is said to be listening. Other research supports the suggestion that when we are said to *imagine* “hearing” speech or music, we are behaving subvocally (Schlinger, 2009). Thus, studies have shown activation in Broca's area and the premotor and motor cortexes when either listening or auditorily imagining (e.g., Paulesu et al., 1993; Zatorre et al., 1996; Halpern and Zatorre, 1999; Rosen et al., 2000; Palmer et al., 2001; Wilson et al., 2004), and support the contention that what we speak of as listening and auditory imagining are more parsimoniously viewed as behaviors, not cognitive processes.

## Conclusion

It is behavior, not cognitive events, which is important for organisms—human and nonhuman—both evolutionarily and in their own lifetimes. Behavior interacts with and adapts to the (i.e., is selected by the) environment; and the nervous system has evolved to support that interaction. Behavior analysis, as a science of behavior in its own right, and not as an indicator of inferred cognitive structures or processes, is best positioned to parsimoniously explain that interaction. Neuroscientists require a cogent theory of behavior to support their search for the neurophysiological correlates of behavior. Thus, behavior analysis can offer both an experimental model based on single-subject research and an elegant theory of behavior that can provide neurophysiologists a non-dualistic road map for understanding the neurophysical correlates of adaptive behavior.

### Conflict of interest statement

The author declares that the research was conducted in the absence of any commercial or financial relationships that could be construed as a potential conflict of interest.
